# Recurrent laryngeal nerve lymph nodes status prediction after neoadjuvant therapy for thoracic esophageal squamous cell carcinoma

**DOI:** 10.1186/s13244-026-02277-6

**Published:** 2026-04-07

**Authors:** Fengze Wu, Chao Luo, Shumin Zhou, Zexue Peng, Guangying Ruan, Xin Yang, Shuqi Li, Wenjie Huang, Lizhi Liu, Jian Zhou, Baodan Liang, Haojiang Li

**Affiliations:** 1 Department of Radiology, Sun Yat-sen University Cancer Center, State Key Laboratory of Oncology in South China, Guangdong Provincial Clinical Research Center for Cancer, Collaborative Innovation Center for Cancer Medicine, Guangdong Key Laboratory of Nasopharyngeal Carcinoma Diagnosis and Therapy, Guangdong Esophageal Cancer Institute, Guangzhou, Guangdong, China; 2 Department of Colorectal Surgery, Sun Yat-sen University Cancer Center, Guangzhou, China; 3 Department of Radiology, Xiangya Changde Hospital, Changde, China; 4 Department of Radiotherapy, Sun Yat-sen University Cancer Center, State Key Laboratory of Oncology in South China, Collaborative Innovation Center for Cancer Medicine, Guangdong Key Laboratory of Nasopharyngeal Carcinoma Diagnosis and Therapy, Guangdong Esophageal Cancer Institute, Guangzhou, Guangdong, China; 5 Department of Radiology, The Third People’s Hospital of Shenzhen, Guangdong, China; 6 South China Hospital, Medical School, Shenzhen University, Shenzhen, China; 7 Department of Radiology, People’s Hospital of Yangjiang, Yangjiang, Guangdong, China

**Keywords:** Esophageal neoplasms, Neoadjuvant therapy, Nomogram, Computed tomography, Chemoradiotherapy

## Abstract

**Objectives:**

Accurately predicting recurrent laryngeal nerve lymph nodes (RLNs) status after neoadjuvant therapy is challenging but essential for efficient dissection of RLNs and for lowering local recurrence rates in patients with esophageal cancer.

**Materials and methods:**

In this retrospective study, 403 patients diagnosed with esophageal squamous cell cancer between 2010 and 2021 were included and randomly divided into training and test cohorts at a ratio of 2:1. Logistic regression and least absolute shrinkage and selection operator (LASSO) regression analyses were conducted to identify significant factors associated with residual RLNs. Multivariable logistic regression analysis was used to develop the final nomogram by integrating clinical factors and pre- and post-treatment CT imaging features. The discriminatory ability of the nomogram was assessed using the area under the receiver operating characteristic (ROC) curve.

**Results:**

The RLN metastatic rate after neoadjuvant therapy was 13.2%. Significant predictors of RLN (+) status post-neoadjuvant therapy identified by LASSO regression analysis included neoadjuvant therapy plan, serum albumin level, long diameter of the primary lesion, baseline necrosis of RLNs, baseline long diameter of RLNs, and short diameter of RLNs post-neoadjuvant treatment. The nomogram demonstrated good discriminative ability, with an area under the ROC curve of 0.856 in the test set. Neoadjuvant chemoradiotherapy was associated with a 70% reduction in the rate of RLN residues compared to neoadjuvant chemotherapy alone.

**Conclusion:**

The nomogram, based on clinical factors and pre- and post-treatment CT imaging features, provides a superior discriminatory ability for predicting the pathological status of RLNs after neoadjuvant chemoradiotherapy.

**Critical relevance statement:**

The nomogram presents a convenient and noninvasive method for evaluating the residual risk of RLNs after treatment for clinicians. It has the potential to provide guidance for a more precise lymph node dissection in patients with low residual risk.

**Key Points:**

- Accurate RLNs status prediction can lower local recurrence rates in esophageal cancer.
- The nomogram can be used for RLN pathological status prediction.
- The nomogram is convenient and noninvasive for the assessment of residual risk of RLNs.

**Graphical Abstract:**

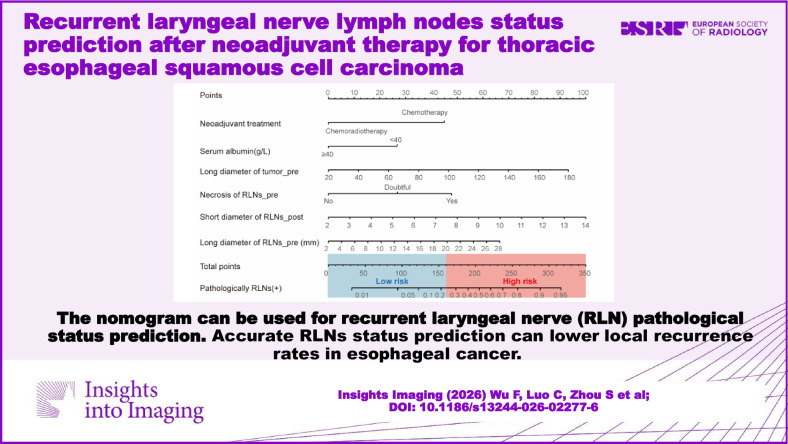

## Introduction

Esophageal cancer ranks seventh in global cancer-related deaths, with 510,716 new cases and 445,129 fatalities in 2022 [1]. Globally, approximately 85% of esophageal cancer cases are attributed to squamous cell carcinoma, with a notably elevated burden in such regions as East Asia and East Africa [2]. Esophageal squamous cell carcinoma (ESCC) frequently metastasizes to the recurrent laryngeal nerve lymph nodes (RLNs) at rates of 20–40% [3]. Residual lymph nodes after neoadjuvant therapy are both an independent prognostic factor for survival and an independent risk factor for recurrence following surgery for esophageal cancer [4, 5]. Effective RLN dissection can reduce local recurrence rates and significantly extend the overall survival in patients with esophageal cancer [6]. However, due to the RLNs’ unique anatomical location, the recurrent laryngeal nerve is susceptible to injury during lymph node dissection. This can lead to complications such as hoarseness, recurrent laryngeal nerve palsy, and lung infections, which not only diminish patient quality of life but also result in poor prognosis in patients with esophageal cancer [7, 8]. Previous studies have suggested that patients with negative RLNs should avoid unnecessary dissection [9]. Accurate RLN status prediction post-neoadjuvant therapy is crucial to avoid unnecessary dissection and ensure effective treatment.

Detecting lymph node metastasis on CT images is challenging but crucial [10]. The incidence of occult RLNs metastasis after neoadjuvant chemoradiotherapy (nCRT) in patients with ESCC remains high at 19.6% (11/56) [11]. The primary factor in defining RLN metastases is the minimal axial diameter of the lymph nodes, usually set at a short diameter of > 10 mm. Nevertheless, studies suggest a smaller short-diameter threshold may be more effective in diagnosing metastatic RLNs [6]. Diagnosing residual RLNs based solely on size or specific indicators has limited accuracy [12, 13]. Previous studies indicate that clinical and primary tumor features can predict RLN metastasis [14, 15]. However, they are not related to lymph nodes, and the responses of the primary tumor and lymph nodes to treatment may not align. Most studies have focused on pre-treatment features, and there are no studies combining pre- and post-treatment features for esophageal cancer. Given that tumors respond variably to distinct treatments, we propose that the lymph node status post-neoadjuvant therapy can be better predicted by considering both pre- and post-treatment characteristics.

Structured reports have become increasingly common in recent years. They are generated using a standardized organizational structure that systematically incorporates all necessary imaging diagnostic information. They play a crucial role as supplementary tools to commonly used diameter standards for assessing tumors and lymph nodes. Structured reports have proven to be effective in the accurate depiction of tumor staging in rectal cancer [16], guidance for the selection of optimal treatment for pancreatic cancer [17], and for nasopharyngeal carcinoma [18]. However, CT-structured studies of esophageal cancer are lacking.

Hence, our goal was to establish models for the prediction of the RLN status post-neoadjuvant therapy by combining the structural characteristics of pre- and post-treatment CT scans.

## Materials and methods

### Patients and data collection

This retrospective study was approved by the Institutional Review Board of Sun Yat-sen University Cancer Center (SYSUCC). Ethical approval was granted by the Institutional Review Board of SYSUCC (approval number: B2021-335-01). Given the retrospective design of this study, the need for informed consent was waived in accordance with the principles of the Declaration of Helsinki, 2013 [19].

**Fig. 1:**
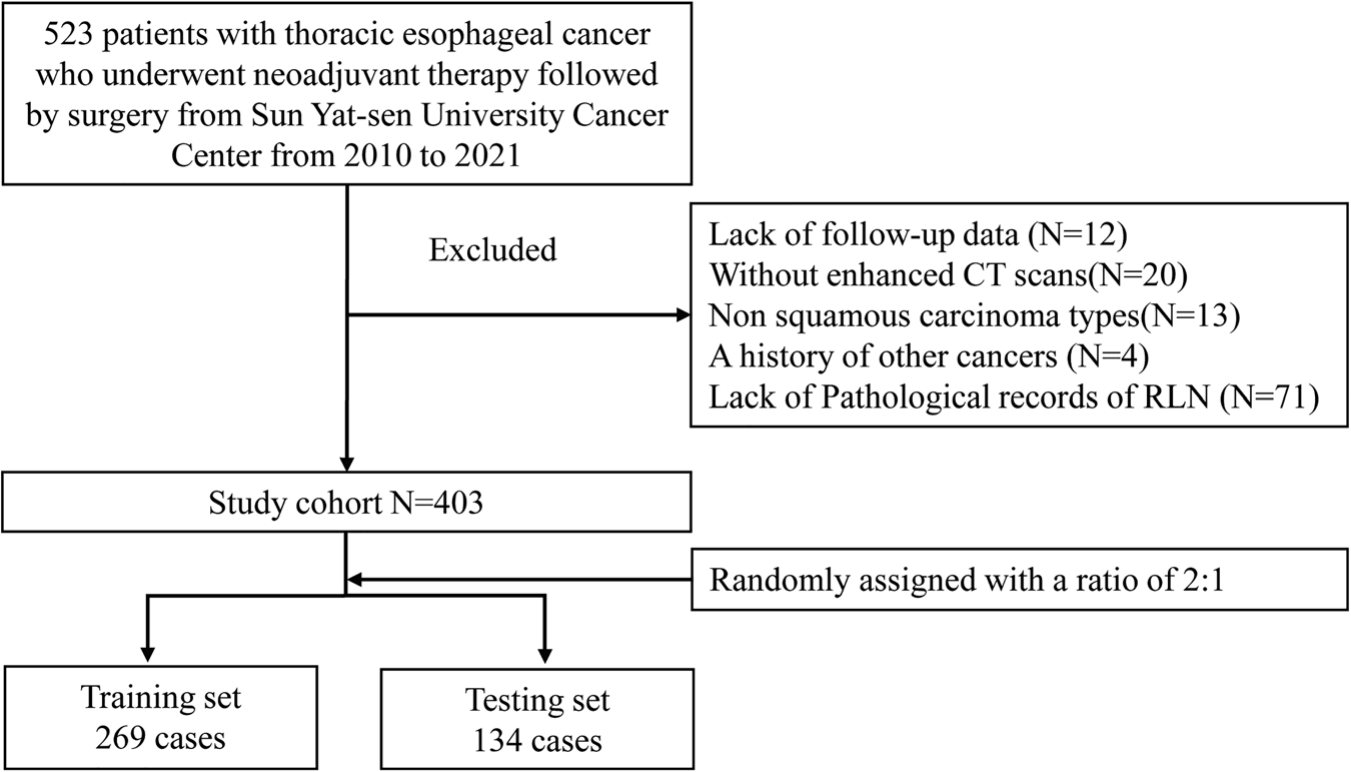
Flowchart of patient inclusion. RLNs, recurrent laryngeal nerve lymph nodes

We conducted a retrospective review of data from 403 eligible patients with locally advanced ESCC (LAESCC) who were treated at the SYSUCC between 2010 and 2021 (Fig. 1). The inclusion criteria were (1) pathologically confirmed thoracic ESCC, (2) radical resection and lymph node dissection following neoadjuvant therapy (chemotherapy or chemoradiotherapy), and (3) without distant metastasis. The exclusion criteria were: (1) lack of follow-up data, (2) without enhanced CT scans, (3) a history of other cancers, (4) multiple primary tumors, and (5) lack of pathological records of RLN.

Clinical text data and medical images were extracted from electronic medical records and picture archiving and communication systems, respectively. Clinical text data included demographic information (sex, age, smoking and alcohol consumption habits, and family history of tumors), baseline hematological indices (lactate dehydrogenase, alkaline phosphatase, serum albumin (ALB), etc.), treatment plans, and pathological data (histological grade, lymph node status, particularly RLNs, and complete response). Medical images included both pre- and post-treatment (neoadjuvant therapy) CT scans.

### Follow-up and endpoint

Patients underwent follow-up for 5 years, with assessments scheduled at 3-month intervals for the initial 2 years and 6-month intervals thereafter. Overall survival (OS) was calculated from the date of surgery to the date of death from any cause. Progression-free survival (PFS) was defined as the duration from surgery until the first recorded instance of disease progression or mortality. The primary endpoint was patient-level pathological residual RLN after neoadjuvant therapy, irrespective of side.

### CT image protocol and assessment

Patients underwent examination using five CT scanners (Discovery CT750 HD, AQUILION TSX-101A, SOMATOM Force, Brilliance iC, and uCT78). Scans covered the region from the thoracic inlet to 2–7 cm below the lower costal margin, adjusted based on lesion extent. Tube voltage was set at 120 kV, with tube current modulation from 100 to 300 mA. The field of view ranged from 400 to 500 mm, and the matrix size was 512 × 512. Slice thickness and interval were set at 5 mm, and thin-slice reconstruction parameters were configured with a thickness of 1.25 mm. For contrast-enhanced scanning, an antecubital vein channel was established before the scan. A high-pressure injector administered a non-ionic iodinated contrast agent at 3.0 mL/s, with arterial and venous phase images acquired at delays of 25 s and 55 s, respectively.

**Fig. 2:**
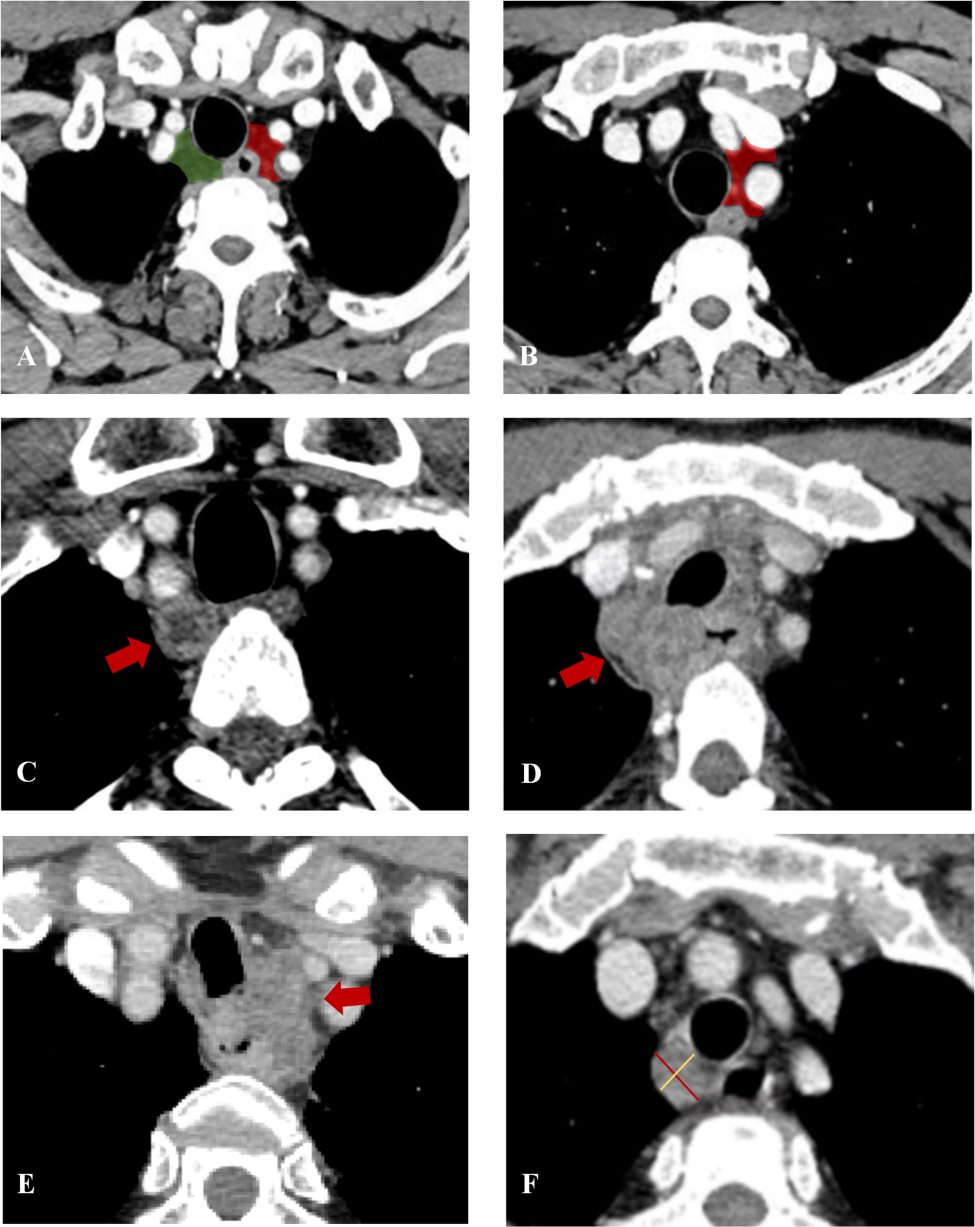
The atlas of RLN stations and examples of the assessment of specific imaging features of RLNs. **A** Left (red area) and right (green area) RLN stations at the level of the thoracic inlet. **B** Left RLN station(red area) at the level above the aortic arch. **C** Necrosis: low-density lesions in the lymph nodes without enhancement. **D** Fusion: The fat space between the lymph nodes disappeared. **E** Extracapsular invasion: lymph node capsule discontinuity. **F** Diameter measurement: the yellow line represents the short diameter, defined as the maximum diameter perpendicular to the long diameter (red line). RLNs, recurrent laryngeal nerve lymph nodes

Based on previous records and examination timing (pre- or post-treatment), CT scans of patients were evaluated using a structured CT report template for primary tumors (Table S1) and RLNs (Table S2). Initially, two senior radiologists (L.H.J. and P.Z.X., both with 10 years of experience in diagnosing digestive system tumors) established consensus by assessing variables in 30 patients collaboratively. A third, more experienced radiologist (L.L.Z., with 18 years of post-training experience), was consulted in case of disagreements. Subsequently, 100 randomly selected patients (approximately 25% of all enrolled patients) were independently evaluated for agreement between their assessments. The remaining patients were evaluated separately by the two radiologists. Specifically, T and N staging followed the 8th edition of the American Joint Committee on Cancer staging system guidelines. The long diameter of the primary tumor was defined as the axial distance between its first and last layers. Regression rate was calculated as follows: Regression rate = (Diameter_post-Diameter_pre) / Diameter_pre × 100%. On CT imaging, the right RLN station is defined based on its anatomical course [20] as the region between the esophagus and the right common carotid artery, superior to the bifurcation of the brachiocephalic artery. Similarly, the left RLN station is defined as the region between the esophagus and the left common carotid artery, cephalad to the inferior border of the aortic arch. Figure 2 provides an atlas of the RLN stations and examples of the assessment of certain imaging features of RLNs. The corresponding changes in RLNs following therapy are illustrated in the Fig. S4.

### Development of prediction models

Patients were randomly assigned to training or testing cohorts at a 2:1 ratio. Variable selection was performed using univariate logistic regression and the least absolute shrinkage and selection operator (LASSO) method. The features retained were subsequently entered into a standard multivariable logistic regression analysis to construct the final combined model. Based on this final combined model, a predictive nomogram was developed. The contrast models included baseline models (incorporating baseline clinical information and primary tumor-related features) and RLN-related models (models based on pre-treatment features, post-treatment features, and regression rate). Model performances were compared across the training and test sets.

### Statistical analysis

Clinical characteristic distribution differences were assessed using Chi–squared or Fisher’s exact test for categorical variables, while the Student’s *t*-test was used for continuous variables. Kappa was used to evaluate inter-observer agreement for categorical variables such as nodal necrosis, and intraclass correlation coefficients (ICC) were utilized for diameter measurements. The combined model was established using LASSO regression. To gauge the models’ discriminative ability, diagnosis accuracy was evaluated using receiver operating characteristic (ROC) and precision-recall (PR) curves, with the area under the curve (AUC) as a performance measure. DeLong’s test was used to compare the AUC values between various models. The calibration curve allowed for a visual assessment of consistency. Furthermore, we conducted a decision curve analysis to evaluate clinical utility, calculating net benefits across different threshold probabilities within the dataset. Additionally, ROC curves were used to determine the optimal cutoff values for RLN diameters. Differences in survival outcomes stratified by predicted RLN status were evaluated using the log-rank test, with curves generated via the Kaplan–Meier method.

Statistical analyses were conducted using R software (version 4.2.2, www.r-project.org/), with packages including stats, glmnet, pROC, ggplot2, rms, and others. Continuous variables were presented as medians with interquartile ranges (IQRs) and categorical variables as frequencies (percentages). All statistical tests were two-tailed, with *p* < 0.05 considered statistically significant.

## Results

### Patient characteristics and consistency analysis

In this retrospective study of 403 patients (Table 1), 332 (82.4%) were male, with a median age of 59 (IQR 54–64) years. Most had moderately or poorly differentiated tumors (87.6%) in the middle and lower parts of the esophagus (86.3%). All patients underwent nCRT (60.5%) or neoadjuvant chemotherapy (39.5%). The median follow-up time for the entire cohort was 46.1 months (range: 3.5 to 157.4 months). During this period, 143 deaths (35.5%) were recorded, with a median time to death of 40.6 months. Disease progression occurred in 153 patients (38.0%), and the median progression-free survival was 46.9 months.

**Table 1:** Distribution of clinical and certain imaging characteristics in training and validation cohorts

Variable	Total	Train cohort	Test cohort	*p*-value*
	*N* = 403	*N* = 269	*N* = 134	
Sex				0.679
Male	332 (82.4%)	220 (81.8%)	112 (83.6%)	
Female	71 (17.6%)	49 (18.2%)	22 (16.4%)	
Age (years)	59 (54–64)	59 (54–64)	59 (54–65)	0.340
ALB (g/L)			0.133	
≥ 40	355 (88.1%)	232 (86.2%)	123 (91.8%)	
< 40	48 (11.9%)	37 (13.8%)	11 (8.2%)	
Alcohol consumption				0.673
No	212 (52.6%)	144 (53.5%)	68 (50.7%)	
Yes	191 (47.4%)	125 (46.5%)	66 (49.3%)	
Smoking				0.669
No	160 (39.7%)	109 (40.5%)	51 (38.1%)	
Yes	243 (60.3%)	160 (59.5%)	83 (61.9%)	
Tumor site^§^				0.913
Upper	55 (13.6%)	36 (13.4%)	19 (14.2%)	
Middle	223 (55.3%)	151 (56.1%)	72 (53.7%)	
Lower	125 (31%)	82 (30.5%)	43 (32.1%)	
T stage^§^				0.283
T2	178 (44.2%)	113 (42%)	65 (48.5%)	
T3	198 (49.1%)	135 (50.2%)	63 (47%)	
T4	27 (6.7%)	21 (7.8%)	6 (4.5%)	
N stage^§^				0.007
N0	96 (23.8%)	51 (19%)	45 (33.6%)	
N1	153 (38%)	104 (38.7%)	49 (36.6%)	
N2	108 (26.8%)	78 (29%)	30 (22.4%)	
N3	46 (11.4%)	36 (13.4%)	10 (7.5%)	
Short diameter of RLNs (mm)^†^	7 (5.5–8.8)	7.4 (5.6–9)	6.7 (5.3–8)	0.043
Differentiation			0.146	
High	50 (12.4%)	33 (12.3%)	17 (12.7%)	
Middle	203 (50.4%)	127 (47.2%)	76 (56.7%)	
Low	150 (37.2%)	109 (40.5%)	41 (30.6%)	
pRLN status			0.877	
Negative	350 (86.8%)	233 (86.6%)	117 (87.3%)	
Positive	53 (13.2%)	36 (13.4%)	17 (12.7%)	

*RLNs* recurrent laryngeal nerve lymph nodes, *p* pathologically

* *p*-values are calculated using Fisher’s exact test or χ^2^ test for categorical variables and *t*-test for continuous variables. Categorical variables are shown with patients’ number (percentage), and continuous variables with median (interquartile range)

^§^ The T and N stage are evaluated with CT according to the 8th edition of AJCC criteria for esophageal cancer

^†^ Evaluation at baseline on axial plane CT

The distribution of sex, age, and most clinical characteristics was well-balanced between the training (269 patients) and test cohorts (134 patients). The test data showed a more primitive N stage based on CT than the training data. There was no significant difference in the frequency of residual RLNs between the two groups (13.4% and 12.7% for the training and validation cohorts, respectively).

Good consistency in imaging feature evaluation was observed among radiologists (Table S3). Kappa values exceeding 0.7 were obtained for categorical variables, whereas ICC values exceeding 0.8 were achieved for continuous variables.

### Feature selection and model construction

**Fig. 3:**
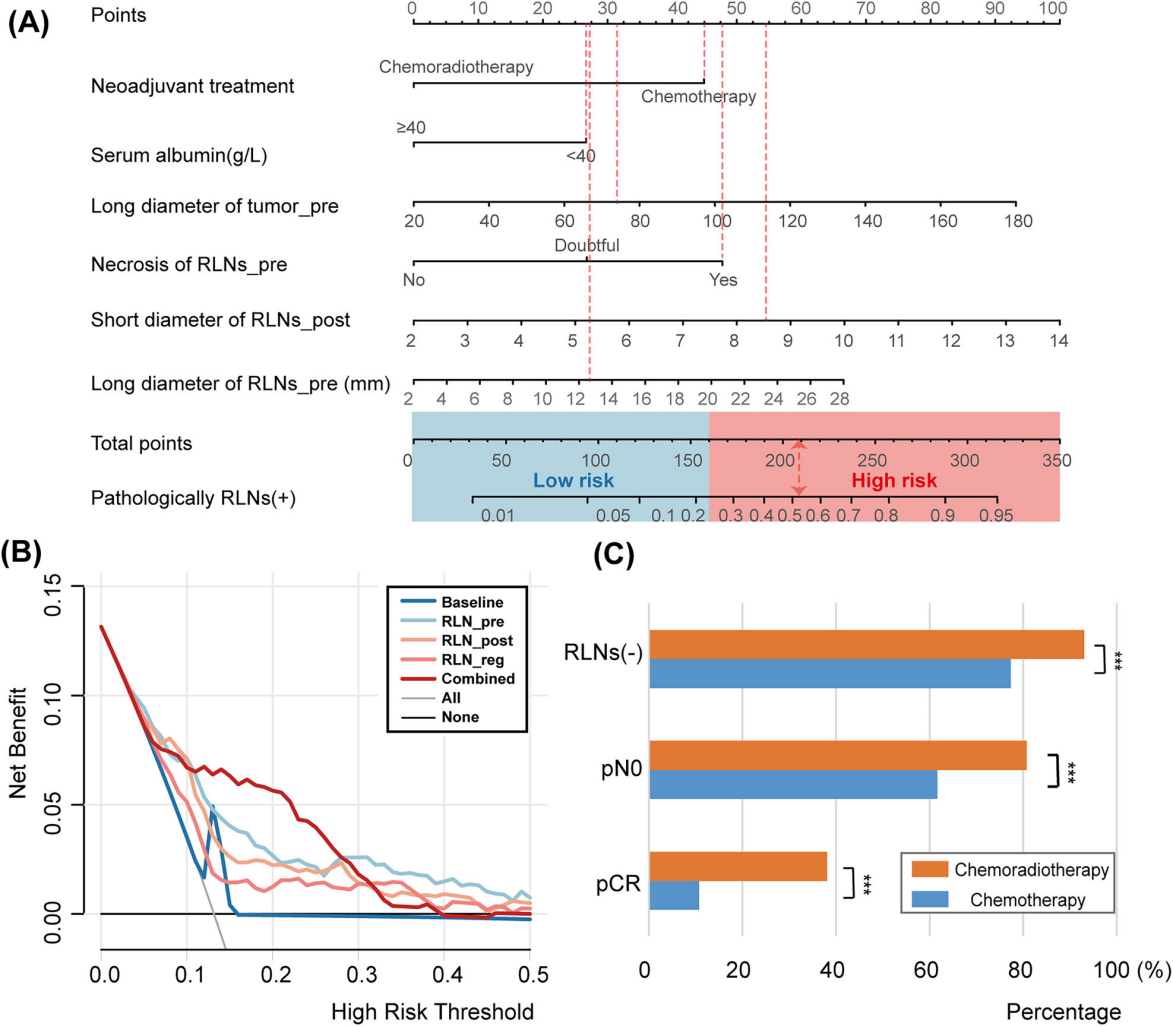
Nomogram of the combined model, decision curve analysis curves of different models, and different pathologically endpoints of different adjuvant therapy plans. **A** Nomograms for predicting pathological RLN status. For example, a patient with the following characteristics: neoadjuvant chemotherapy, albumin < 40 g/L, primary tumor 74 mm, RLN necrosis, pre- and post-neoadjuvant RLN diameters were 12.6 mm and 8.6 mm (red dashed line). The calculated risk score is 225.7, and this patient was confirmed to have positive RLNs. **B** Decision curve analysis curves of different models in the whole cohort: The *X*-axis represents the probability of the pathological RLN status, ranging from 0 to 50%. The *Y*-axis shows the net benefit. The black line indicates that no pathological RLNs (+) occurred in all patients. The gray line represents the assumption that all patients are pathologically RLNs (+). **C** The distribution of different pathological outcomes between patients who received two neoadjuvant therapies (*** *p* < 0.001, using Pearson’s Chi–squared test). RLNs, recurrent laryngeal nerve lymph nodes; pCR, pathologically complete response

Results of the univariate logistic regression analysis in the training set identified alcohol consumption, ALB, and neoadjuvant therapy plans as significant clinical factors related to RLN residues. Additionally, nine RLN-related features and one primary tumor-related feature were identified (Table S4). Multivariate logistic regression analysis was used to establish a baseline model using the significant factors determined in the univariate analysis of baseline clinical data and primary tumor imaging features (Table 2). Similarly, the RLN-related models at various treatment stages are presented in Table S5. LASSO regression analysis was performed based on the significant factors obtained in the univariate analysis, and the features’ LASSO coefficient profiles are depicted in Fig. S1. Subsequently, a combined model was established using multivariate logistic regression analysis (Table 2). Furthermore, a nomogram was developed for a more accurate prediction of pathologically positive RLN status, incorporating independent factors identified in the multivariate logistic regression analysis (Fig. 3A). The nomogram revealed that individuals with total points exceeding 160 were more likely to have pathologically positive RLNs, with a predictive range from 0.01 to 0.95.

**Table 2:** Details of baseline and final combined model

Variable	Multivariate analysis with stepwise		
	Coef	OR (95% CI)	*p*-value*
**Baseline model**^§^			
Alcohol consumption			
No		1 (Ref)	
Yes	0.81	2.25 (1.05–4.98)	0.040
Neoadjuvant therapy plan			
Chemotherapy		1 (Ref)	
Chemoradiotherapy	−1.46	0.23 (0.10–0.50)	0.000
Serum albumin (g/L)			
≥ 40		1 (Ref)	
< 40	0.76	2.15 (0.85–5.17)	0.095
Long diameter of tumor (mm)	0.02	1.02 (1.00–1.03)	0.028
**Combined model**^§^			
Neoadjuvant therapy mode			
Chemotherapy		1 (Ref)	
Chemoradiotherapy	−1.19	0.30 (0.12–0.70)	0.007
ALB (g/L)			
≥ 40		1 (Ref)	
< 40	0.71	2.03 (0.71–5.48)	0.171
Long diameter of tumor (mm)^&^	0.02	1.02 (1.00–1.03)	0.050
Necrosis of RLNs_pre^†^			
None		1 (Ref)	
Possible yes	0.71	2.04 (0.71–5.58)	0.174
Yes	1.27	3.55 (1.25–10.04)	0.016
Short diameter of RLNs_post (mm)^†^	0.22	1.25 (1.03–1.53)	0.029
Long diameter of RLNs_pre (mm)^†^	0.07	1.07 (0.95–1.21)	0.278

*RLNs* recurrent laryngeal nerve lymph nodes, *OR* odds ratio, *CI* confidence interval, *ALB* serum albumin

* *p*-values are calculated using multivariate logistic regression

^§^ The baseline model is developed using clinical and tumor-related variables related to residual RLNs, whereas the combined model incorporates clinical, tumor, and RLNs-related variables associated with residual RLNs (refer to Table S2)

^&^ The long diameter of the primary tumor is defined as the distance between the first and last layers of the tumor in the axial position

^†^ Pre and post: Evaluation before/after receiving neoadjuvant therapy; diameters of RLNs were evaluated on the axial plane CT

### Model performance

**Fig. 4:**
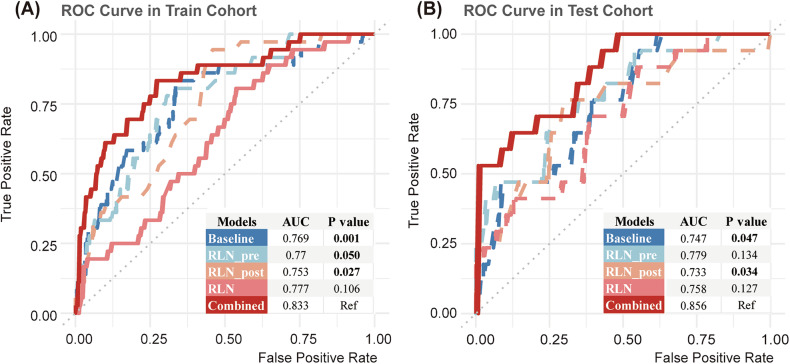
Receiver operating characteristic of different models. Receiver operating characteristic curves of different models in the train (**A**) and test cohort (**B**): more detailed performance information of models is available in Table S6. RLNs, recurrent laryngeal nerve lymph nodes; ROC, receiver operating characteristic; AUC, area under the curve; Ref, reference

The nomogram (AUC = 0.856) demonstrated superior discriminative ability compared with the baseline model (AUC = 0.747) in the ROC curves for the test set (p = 0.047; Fig. 4). Table S6 provides the detailed parameters for model performance. The nomogram outperformed the baseline in PR curves (Fig. S2), with an AUC of 0.514 in the test set. Calibration curves (Fig. S3) showed better alignment with model predictions for the combined model as opposed to the baseline model. For a comprehensive assessment of the clinical utility, a decision curve analysis (Fig. 3B) was conducted for the baseline, RLN-related, and combined models. The combined model offered clinical benefits when the risk ratio was less than 0.4, consistently outperforming other models. In contrast, the baseline model exhibited benefits within a limited range of risk ratios.

### Variable importance analysis in the models

Patients who underwent chemoradiotherapy experienced a 70% decrease in the rate of residual RLNs compared with those who received chemotherapy alone. Further examination of the pathological status (Fig. 3C) revealed that patients who underwent nCRT tended to achieve a pathologically negative RLN, N0, and pathologically complete response (pCR, all *p* < 0.001). The long diameter of RLNs and necrosis in RLNs at baseline and the short diameter of RLNs after treatment were identified as valuable factors related to RLN status. Multivariate analysis identified necrosis of RLNs at baseline and the short diameter of RLNs after treatment as significant independent factors. After adjustment with these factors and confounding variables (alcohol consumption, neoadjuvant therapy plan, and long diameter of tumor), previously important factors (those with *p* < 0.05 after adjustment for confounding factors) became non-significant (all *p* > 0.05, Table S7).

**Fig. 5:**
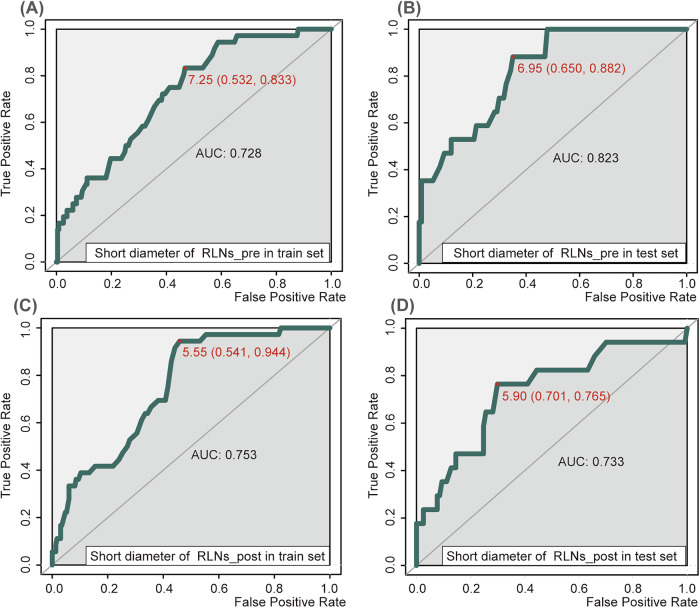
Receiver-operating characteristic curves of different diameters. **A**, **B** ROC curves for the short diameter of pre-treatment RLNs in the train (**A**) and test cohort (**B**). **C**, **D** ROC curves for the short diameter of post-treatment RLNs in the train (**C**) and test cohort (**D**). “Pre” and “post” represent evaluations conducted before and after receiving neoadjuvant therapy, respectively; ROC, receiver operating characteristic; AUC, area under the curve;  RLNs, recurrent laryngeal nerve lymph nodes

Based on the maximum Youden’s index, the optimal cutoff values for the short diameter of RLNs pre- and post-treatment were 7 mm (7.25 mm in the training set, 6.95 mm in the test set) and 6 mm (5.55 mm in the training set, 5.90 mm in the test set), respectively, thus remaining relatively (Fig. 5).

### Prognostic Value of predicted RLN status

Based on the model-predicted RLN status, patients were stratified into two subgroups: Predicted RLN+ (32.8%, 132/403) and Predicted RLN− (67.2%, 271/403). Kaplan–Meier curves for 5-year OS and PFS demonstrated a statistically significant difference between the subgroups (Fig. S5). This result indicates that patients classified as Predicted RLN+ were associated with a poorer prognosis.

## Discussion

In this retrospective study, our nomogram, integrating clinical factors with pre- and post-treatment CT imaging features of RLNs, demonstrated superior discriminatory ability for the prediction of pathologically positive RLN status compared to the baseline and RLN-related models. This was observed in both the training (AUC of ROC curve: 0.833) and test sets (AUC of ROC curve: 0.856). The nomogram incorporated a neoadjuvant treatment plan, serum ALB, long tumor diameter, necrosis, and RLN diameter. To our knowledge, this study is the first to establish a visual model for predicting RLN status in patients with LAESCC after neoadjuvant therapy by integrating clinical risk characteristics with pre- and post-treatment CT features of the primary tumor and RLNs. These findings hold potential value for predicting RLN status after neoadjuvant treatment.

Neoadjuvant therapy combined with surgical resection of primary tumors and potentially metastatic lymph nodes is the optimal treatment for LAESCC [21]. Considering the importance of RLNs as sentinel lymph nodes [22] and their unique anatomy, predicting RLN status after neoadjuvant therapy is crucial for minimizing side effects and preserving efficacy. Current RLN status determination mainly relies on postoperative specimens, causing significant pain and substantial delays in patients without residual RLNs. Lymph node diameter in medical imaging is a key factor [23]; clinically, a diameter exceeding 10 mm indicates positivity. However, some studies suggest a shorter standard diameter should be used [24, 25]. However, no consensus exists on this standard. Accurate prediction of post-treatment RLN status can guide a more selective surgical approach, omitting RLNs and cervical lymph node dissection in patients with low-risk residual RLNs [11]. Recent studies have aimed to create preoperative prediction models for RLNs. Yan et al established a nomogram [15] for predicting RLN status in patients with ESCC, incorporating clinical T staging, biopsy tumor differentiation, preoperative carcinoembryonic antigen, and endoscopic tumor length. However, the imaging features of RLNs were not considered. Chen et al developed a nomogram to predict left and right RLN status using tumor location, T stage, short and long RLN diameters, and preoperative biochemical blood characteristics [14]. Nevertheless, no study has simultaneously investigated imaging features before and after treatment to predict the RLN status after neoadjuvant chemotherapy.

Our study integrated three clinical factors—neoadjuvant therapy approach, alcohol consumption, and ALB levels—into a predictive nomogram. Neoadjuvant therapy typically involves chemotherapy and chemoradiotherapy, with research favoring the higher pCR of chemoradiotherapy and pathological LN-negative rates [26]. Thus, considering the neoadjuvant therapy approach as a risk factor for post-treatment RLN status is justified. Alcohol consumption, a known risk factor for upper digestive tract tumors [27], has been linked to metastasis in various cancers. Our study suggests that alcohol consumption may increase the risk of residual RLNs after neoadjuvant therapy. ALB, an essential nutritional assessment indicator, reflects nutritional status, liver function, and protein consumption [28]. Lower ALB levels may impact treatment response, potentially leading to an incomplete response of lymph nodes to treatment [29]. Therefore, we hypothesized that low ALB levels influence treatment outcomes, thereby contributing to an increased risk of residual RLNs after therapy.

Neoadjuvant therapy followed by radical esophageal cancer resection is preferred for patients with LAESCC [30, 31], thereby significantly improving prognosis [32]. Han et al found that patients who received nCRT had higher 3-year survival, R0 resection, and pCR, and lower local recurrence and distant metastasis rates but no increase in 5-year survival than those who received neoadjuvant chemotherapy [33], which is recommended in Japanese guidelines [34]. The preferred neoadjuvant therapy for patients with LAESCC remains controversial. In our study, we found that patients who received nCRT had lower RLN positivity rates and higher rates of N0 and pCR, indicating that nCRT should be recommended for LAESCC with potential RLN metastasis after neoadjuvant treatment.

The primary independent imaging feature of the pre-treatment primary tumor was its length, associated with the risk of residual RLNs after treatment. Similar findings were reported by Yan et al [15], suggesting that an endoscopic tumor length exceeding 3 cm increased the likelihood of RLN metastasis. Gaur et al [35] supported the predictive value of clinical tumor length for pathologically positive lymph nodes, considering it to be an independent predictor of lymph node metastasis. However, the underlying reason for this association is not yet fully understood. Hou et al [36] suggest that increased tumor length creates a larger interface and greater opportunity for lymphatic engagement. We speculate that when the primary tumor exceeds a certain length, the increased area involved in the submucosal lymphatic network may increase the probability of lymph node micro metastasis, thereby increasing the likelihood of lymph node residue despite the same treatment.

Structural RLN characteristics included in the final model were pre-treatment necrosis, pre-treatment long diameter, and post-treatment short diameter. Post-treatment necrosis may signify treatment response leading to tumor cell death, whereas pre-treatment necrosis, a lymph node metastasis (LNM) characteristic, indicates higher tumor load [37]. Although there was a correlation between the long and short lymph node diameters, the pre-treatment long diameter was significant in the multivariate logistic regression and was therefore included in the model. Due to limitations in the diagnostic criteria of short diameters for LNM, some studies have incorporated both long and short diameters of lymph nodes into preoperative models for predicting lymph node metastasis [38]. Regardless of the diameter, both pre- and post-treatment imaging characteristics of the RLNs were collected in our study. Our study suggests that a short post-treatment diameter better indicates RLN status after treatment than the pre-treatment diameter. Based on the maximum Youden’s index in the ROC curve, the cutoff value was 5.55 mm, signifying a low residual risk when the short post-treatment diameter was < 5.55 mm. This underscores the importance of emphasizing imaging assessments before and after treatment.

Our study had some limitations. First, being a retrospective study, it was susceptible to selection bias. However, efforts were made to include consecutive eligible patients to minimize this bias. Second, due to the clustered distribution of the recurrent laryngeal nerve along the tracheoesophageal sulcus, surgeons could not detect the exact location of the resected RLNs. Consequently, it is challenging to precisely match CT findings with the surgically resected tissue. Therefore, our evaluation only considered the largest lymph node in the recurrent laryngeal region rather than individual RLNs. Third, the study population was derived from a single center, and the generalizability and reproducibility of our model should be assessed with a larger sample from multiple centers as an external validation cohort. Fourth, the sample sizes in left RLN involvement were small; the limited sample size of cases with left RLN involvement precluded a meaningful analysis of nerve involvement.

In summary, we developed a nomogram incorporating clinical features and pre- and post-treatment CT structural reports of primary tumors and RLNs. This nomogram proved to be an effective tool for the individualized prediction of RLN status in patients with locally advanced esophageal cancer following neoadjuvant therapy. For clinicians, the nomogram offers a convenient and noninvasive means of assessing the residual risk of RLNs after treatment. It may guide more targeted lymph node dissection in patients with low residual risk, potentially reducing surgical complications and the risk of recurrent laryngeal nerve injury. Additionally, our study revealed that nCRT may reduce the risk of residual RLNs compared with chemotherapy alone.

## Supplementary information

ELECTRONIC SUPPLEMENTARY MATERIAL

## Data Availability

The data that support the findings of this study are available from the corresponding author upon reasonable request.

## Abbreviations

ALB: Albumin
AUC: Area under the curve
ESCC: Esophageal squamous cell carcinoma
IQRs: Interquartile ranges
ICC: Intraclass correlation coefficients
LASSO: Least absolute shrinkage and selection operator regression models
LAESCC: Locally advanced ESCC
LNM: Lymph node metastasis
nCRT: Neoadjuvant chemoradiotherapy
OS: Overall survival
pCR: Pathologically complete response
PR: Precision-recall
PFS: Progression-free survival
ROC: Receiver operating characteristic
RLNs: Recurrent laryngeal nerve lymph nodes
SYSUCC: Sun Yat-sen University Cancer Center
