# Supplementary material for: Recurrent laryngeal nerve lymph nodes status prediction after neoadjuvant therapy for thoracic esophageal squamous cell carcinoma
Source: Insights Imaging. 2026 Apr 7;17:95. doi: 10.1186/s13244-026-02277-6 (PMC13057039; doi:10.1186/s13244-026-02277-6)
Supplement: Supplementary file 1 — ELECTRONIC SUPPLEMENTARY MATERIAL [file 13244_2026_2277_MOESM1_ESM.pdf]

Recurrent Laryngeal Nerve Lymph Nodes Status Prediction after Neoadjuvant Therapy for Thoracic Esophageal Squamous Cell Carcinoma

ELECTRONIC SUPPLEMENTARY MATERIAL

Supplementary Material

Table S1. Structured computed tomography report template with specific details related to the primary tumor

Primary lesion-related features		
1. Location	Upper	
	Middle	
	Lower	
2. Diameter (cm)	Wall thickness	
	Maximum	
	Maximum (avoid cavities)	
	Length	
3. Enhancement	Homogeneous	
	Possible heterogeneous	
	Heterogeneous	
4. Adjacent structure	Mediastinum	
	Pleura	
	Pericardium	
	Azygos vein	
	Diaphragm	
	Peritoneum	
	Aorta	
	Centrum	
	Trachea	

Table S2. Structured computed tomography report template with specific details related to RLNs

RLNs related features		Left	Right
1. Location			
2. Axial diameter	Short diameter		
	Long diameter		
3. Central necrosis	Negative		
	Possible		
	Positive		
4. Fusion	Negative		
	Possible		
	Positive		
5. Extracapsular invasion	Negative		
	Possible		
	Positive		
6. Enhancement	Homogeneous		
	Possible heterogeneous		
	Heterogeneous		

Abbreviations: RLNs, recurrent laryngeal nerve lymph nodes

Table S3. Consistency analysis of some imaging features assessed by two different radiologists

Variable	Consistency test	Value
**Tumor site**	kappa	0.934
**T stage**	kappa	0.700
**N stage**	kappa	0.820
**RLNs fusion**	kappa	0.786
**RLNs necrosis**	kappa	0.813
**RLNs enhancement**	kappa	0.881
**Short diameter of RLNs**	ICC	0.877
**Long diameter of RLNs**	ICC	0.918
**Long diameter of tumor**	ICC	0.923

Abbreviations: RLNs, recurrent laryngeal nerve lymph nodes; ICC, intraclass correlation coefficients

Table S4. Results of univariate analysis of variables in the training set

Variable Classification	Variable§	Level	p value*
**Clinical**	Alcohol consumption	N/Y	0.027
	Neoadjuvant therapy	Chemotherapy/Chemoradiotherapy	<0.001
	Serum albumin	<40 / ⩾ 40g/L	0.011
**Tumor**	mrN stage	N0/N1/N2/N3	0.042
	Long diameter of tumor_pre	Continuous	0.008
	Max diameter* of tumor_pre	Continuous	0.042
**RLNs_pre†**	Extracapsular invasion of RLNs	N/P/Y	0.032
	Necrosis of RLNs	N/P/Y	<0.001
	Enhancement of RLNs	Homo/Possible hetero/heterogeneous	<0.001
	Short diameter of RLNs	Continuous	<0.001
	Long diameter of RLNs	Continuous	<0.001
**RLNs_post†**	Necrosis of RLNs	N/D/Y	0.003
	Enhancement of RLNs	Homo/Possible hetero/heterogeneous	0.005
	Short diameter of RLNs	Continuous	<0.001
	Long diameter of RLNs	Continuous	<0.001

Notes: *: p values are calculated using univariate logistic regression in train cohort.

§: Only variables with single factor significance are enumerated here.

†: Pre and post: Evaluation before/after receiving neoadjuvant therapy.

Abbreviations: RLNs, recurrent laryngeal nerve lymph nodes; N, no; Y, yes; P, possible yes

*: The max axial diameter with the exclusion of cavities.

Table S5. Details of the RLN-related models

Models (num)	Multivariate analysis with stepwise		
	**Coef**	**OR (95%CI)**	**p value**
**Model of variables before neoadjuvant therapy**			
Necrosis_pre			
None		Ref	
Possible yes	0.8	2.23 (0.82–5.74)	0.102
Yes	1.27	3.57 (1.36–9.27)	0.009
Long diameter_pre	0.15	1.16 (1.05–1.28)	0.004
**Model of variables after neoadjuvant therapy**			
Short axis_post	0.38	1.46 (1.26–1.72)	<0.001
**Model of variables before and after neoadjuvant therapy**			
Necrosis_pre			
None		Ref	
Possible yes	0.74	2.10 (0.76–5.52)	0.138
Yes	1.57	4.80 (1.67–13.82)	0.003
Short diameter_pre	0.36	1.44 (1.22–1.72)	<0.001
Short diameter difference	-0.37	0.69 (0.54–0.86)	0.002

Abbreviations: RLNs, recurrent laryngeal nerve lymph nodes; OR, odds ratio; CI: confidence interval

Table S6. Performance of different models

Model	Train cohort					Test cohort				
	**AUC**	**p value***	**Sen**	**Sep**	**ACC**	**AUC**	**p value***	**Sen**	**Sep**	**ACC**
**Baseline model**	0.769	**0.001**	0.83	0.67	0.69	0.747	**0.047**	0.65	0.67	0.66
**RLN related model**										
**RLN_pre model**	0.770	0.050	0.81	0.69	0.7	0.779	0.134	0.53	**0.77**	0.74
**RLN_post model**	0.753	0.027	0.94	0.54	0.59	0.733	**0.034**	0.76	0.65	0.66
**RLN_reg model**	0.777	0.106	0.81	0.46	0.51	0.758	0.127	0.71	0.56	0.57
**Combined model**	**0.833**	Ref	0.83	**0.73**	**0.74**	0.856	Ref	0.71	**0.77**	**0.76**

Notes: *: p values are calculated using Delong test.

Abbreviations: RLNs, recurrent laryngeal nerve lymph nodes; ACC, accuracy

Table S7. Testing for independent RLN-related variables

Variable	Adjustment univariate analysis [a]		Adjustment univariate analysis [b]	
	OR (95% CI)	p value	OR (95% CI)	p value
**Necrosis_pre**				
None				
Doubt	2.75	**0.040**	2.16	0.138
Yes	5.52	**0.000**	4.23	**0.004**
**Homo-enhancement_pre**				
None				
Doubt	1.68	0.304	0.63	0.538
Yes	3.70	**0.005**	0.18	0.156
**Necrosis_post**				
None				
Doubt	2.84	**0.022**	1.06	0.915
Yes	3.75	**0.024**	0.90	0.894
**homo-enhancement_post**				
None				
Doubt	2.13	0.091	0.70	0.609
Yes	3.00	**0.026**	0.56	0.494
**Short diameter of RLNs_pre**	1.21	**0.001**	1.06	0.456
**Long diameter of RLNs_pre**	1.18	**0.001**	1.07	0.252
**Short diameter of RLNs_post**	1.36	**0.000**	1.29	**0.006**
**Long diameter of RLNs_post**	1.13	**0.030**	0.86	0.159

Notes: [a]: Univariate analysis adjusted with common confounding variables (drink, Neoadjuvant therapy plan and long diameter of tumor).

[b]: Univariate analysis adjusted with confounding variables (necrosis of RLNs_pre and short diameter of RLNs_post, besides confounding variables mentioned in note a).

Abbreviations: RLNs, recurrent laryngeal nerve lymph nodes; OR, odds ratio; CI, confidence interval

Figure S1. Process of variable screening via LASSO

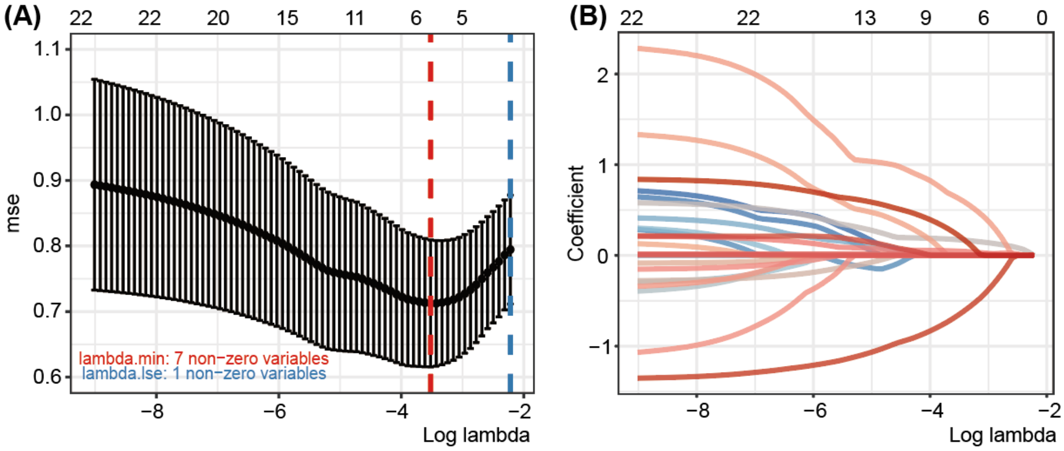

Notes: (A) The curve depicts the change in partial likelihood deviance with log(λ), where λ is the tuning parameter for the LASSO model. The selection of the optimal parameter (λ) in the LASSO model is performed using 10-fold cross-validation. The red and blue vertical lines correspond to the minimum partial likelihood deviance and one standard error from the minimum partial likelihood deviance, respectively. The optimal λ value, corresponding to the minimum partial likelihood deviance, is chosen, and at this point, log(λ) equals -3.521. LASSO Regression path plot (B) illustrates the changes in the regression coefficients of each variable under different values of the penalty parameter λ. Categorical variables were converted into dummy variables (reference-based) for LASSO regression, thus the total number of variables in the plot exceeds that listed in tables.

Abbreviations: LASSO, least absolute shrinkage and selection operator

Figure S2. Precision-recall curves of different models

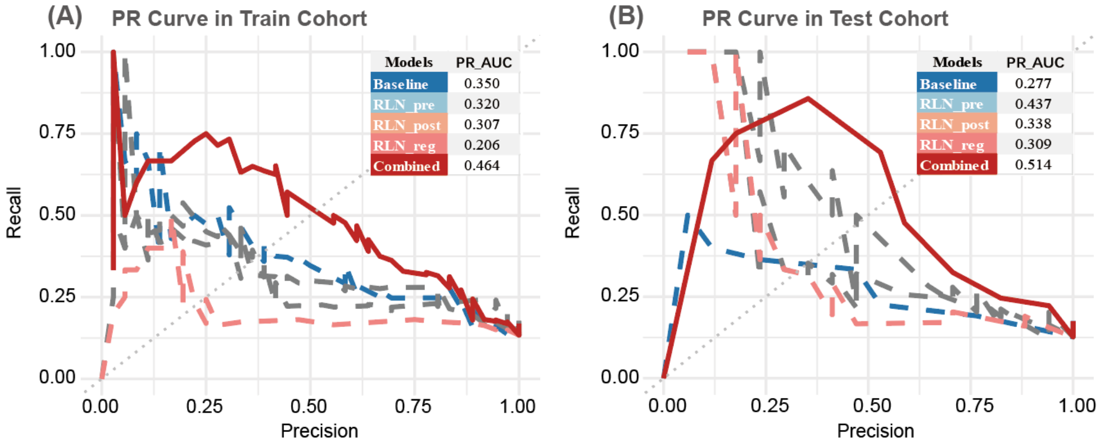

Notes: Precision-recall curves of different models in the primary (A) and test cohort (B).

Abbreviations: RLNs, recurrent laryngeal nerve lymph nodes. PR, Precision-recall; AUC, area under the curve; Ref, reference.

Figure S3. Calibration curves of different models

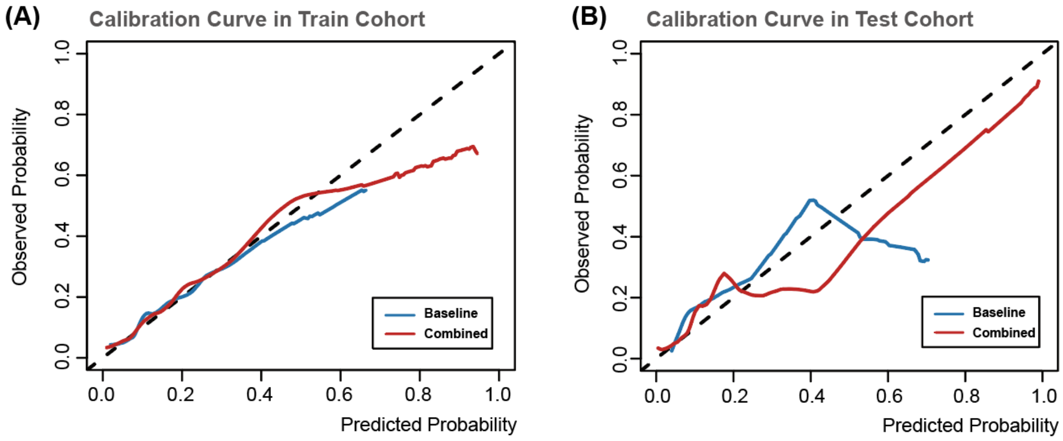

Notes: Calibration curves of different models in the primary (A) and test cohort (B).

Figure S4. Case examples of RLNs Changes pre and post therapy

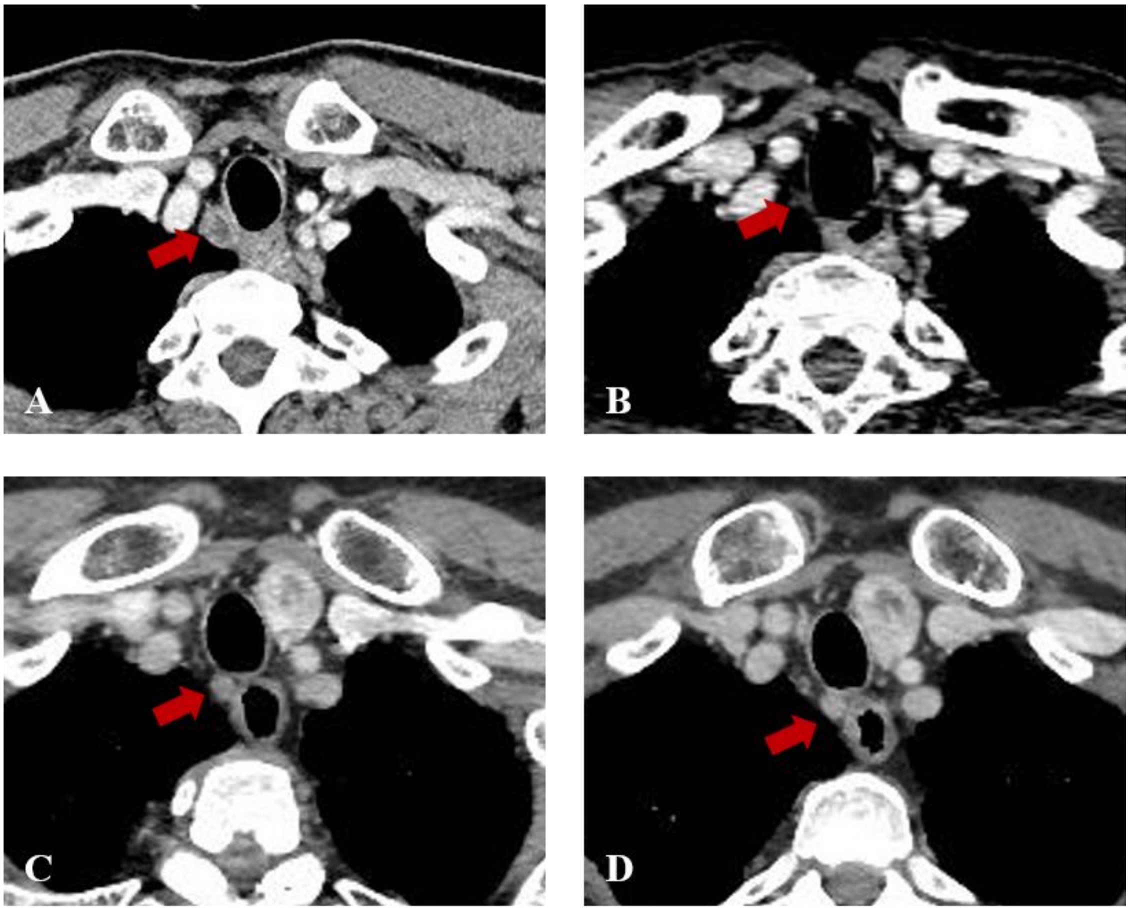

Notes: Axial CT images from a 53-year-old male patient with ESCC, the baseline scan (A) identified an enlarged right RLN with central necrosis (arrow). Following three cycles of concurrent chemoradiotherapy, the follow-up scan (B) demonstrated marked regression of the node. Subsequent pathological examination of the dissected right RLN stations revealed fibrotic tissue only; CT images from a 62-year-old male patient with ESCC had a right RLN with a short-axis diameter of 6 mm (arrow) identified on the initial CT scan (C). Follow-up imaging after three cycles of neoadjuvant chemotherapy (D) revealed no significant change in the node, which was later pathologically confirmed as a pathological lymph node

Abbreviations: ESCC, esophageal squamous cell carcinoma; RLN, Recurrent laryngeal nerve lymph node.

Figure S5. Kaplan-Meier curves of the final model in the total cohort for OS and PFS

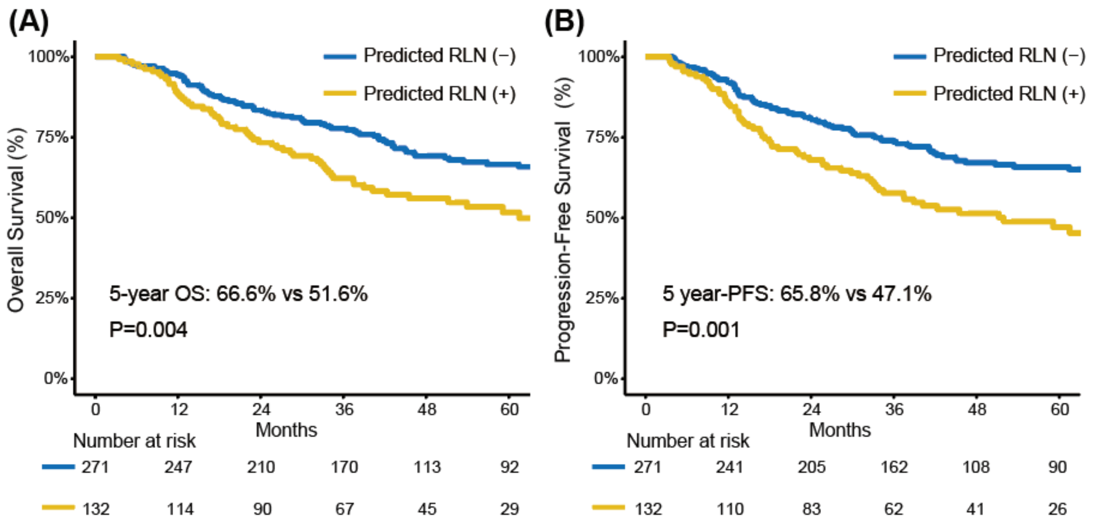

Abbreviations: RLN, recurrent laryngeal nerve lymph node; OS: overall survival; PFS, progression-free survival.
